# Qualitative and Quantitative Outcomes of a 1:1 Multidisciplinary Weight Management Clinic

**DOI:** 10.3390/healthcare3020429

**Published:** 2015-06-10

**Authors:** Desley Turner, Nadim Haboubi

**Affiliations:** Aneurin Bevan Multidisciplinary Specialist Weight Management Centre, Ysbyty Aneurin Bevan, Ebbw Vale NP23 8XE, UK; E-Mail: desleyturner@doctors.org.uk

**Keywords:** obesity, weight management, BMI

## Abstract

*Background*: Obesity management in Wales includes the provision of a 1:1 Multidisciplinary Weight Management Clinic (MDWMC). Strategic management of obesity in Wales is guided by The All Wales Obesity Pathway and recommends MDWMCs for people with obesity who have one or more co-morbidities and who have tried several interventions without success, or who have complex emotional relationships with food. No known previous studies have included a qualitative evaluation of a MDWMC. *Objectives*: To conduct a service evaluation of a 1:1 Multi-disciplinary Weight Management Clinic to evaluate associated physiological benefits and qualitative data about the service. *Methods*: Semi-structured interviews were conducted with 180 patients attending the MDWMC at Aneurin Bevan Hospital, Ebbw Vale, Wales. *Results*: The MDWMC supports weight loss with 95% of patients reporting loss. For those for whom baseline data was available 73% lost at least 5% of initial body weight. Eighty-eight percent of patients prefer individual appointments and over 90% of patients who see each team member find consultations useful. Sixty-nine percent of patients report improved health mainly due to a decrease in obesity-related symptoms, and of patients taking obesity-related medication 48% report a reduction in dose of medication for asthma, 42% report a reduction in dose of antidepressants, and 36% report a reduction in dose for medication for diabetes. Of employed patients, 30% report a reduction of days taken off work due to sickness. Ninety-six percent of patients would recommend the clinic to others. *Conclusions:* A 1:1 Multi-disciplinary Weight Management Clinic provides value in reducing obesity and symptoms of obesity-related diseases. It also is a treatment choice favoured by patients.

## 1. Introduction

### 1.1. The Obesity Epidemic

When discussing the issue of obesity “it is said that we are facing a public health problem that is comparable to climate change both in its scale and complexity” [1]. Globally, obesity has doubled from 1980 to 2008, more than 1.4 billion adults, 20 and older, were overweight: of these, over 200 million men and nearly 300 million women were obese [2]. Figures forecasting the problem in the UK predict that, if current trends persist, 40% of the population could be classified as obese by 2025 [3]. The most prominent serious physiological health consequences of being obese include increased risks of developing type 2 diabetes, cardiovascular disease, musculoskeletal disorders, such as osteoarthritis, and many types of cancer [4]. Psychosocially, persons can be exposed to discrimination and prejudice, and being overweight has an association with depression [5].

The costs of the obesity epidemic are significant: in the UK, the burden on the NHS was estimated to be £4.2 billion in 2007, and the rise in costs attributable to elevated BMI is predicted to be £9.7 billion by 2050 [3]. Managing the issue effectively is becoming increasingly important and any treatment drawing on limited funds must be shown to deliver value.

### 1.2. The Problem—Wales

In Wales, 57% of adults are classified as overweight or obese, including 22% in the obese category [6]. While the increasing rate parallels that in most other Western countries, Wales faces a regionally specific future given that 21% of boys and 18% of girls aged 15 are overweight or obese: this is one of the highest percentages in the Western world, and substantially higher than in Scotland and England [7]. The current cost of obesity to the NHS in Wales is estimated at over £73 million, and “in September 2008, between more than £1.40 million and £1.65 million was spent each week treating diseases resulting from obesity” [7].

### 1.3. The Problem—Aneurin Bevan Health Board

Aneurin Bevan Health Board is an area in South East Wales covered by NHS Wales. It covers some of the areas of highest unemployment claimants, most notably in Blaenau Gwent at 7.9% and Torfaen at 5.4% [8]. The health board’s population includes areas with the highest levels of obesity in the UK [9], levels that are above the 22% Welsh national average at 26%–27% in November 2012, and are likely to be exacerbated by unemployment and social deprivation. Blaenau Gwent Local Authority received funding from the Welsh Assembly Government to address obesity locally, and one of the services implemented was the 1:1 Multidisciplinary Weight Management Clinic (MDWMC), which is of interest in this report. The MDWMC in Aneurin Bevan Health Board is the only of its kind running in Wales.

### 1.4. The All Wales Obesity Pathway

Strategic plans to tackle obesity in Wales follow the All Wales Obesity Pathway [1]. Launched by the Welsh Assembly Government in 2009, it relates to guidelines published by NICE in 2006 [10]. The Pathway “is a guidance tool for NHS Health Boards to inform which services should be available to support the management of obesity” [1]. In a broad sense, Level 1 interventions include community-based prevention and self-referral, Level 2 includes community and primary care weight management services, Level 3 involves specialist weight management services, pharmacological interventions, and Level 4 includes specialist medical and surgical services (Appendix A).

### 1.5. Level 3 Service

More specifically, Level 3 aims to provide specialist weight management services to adults who have one or more co-morbidities, and who have tried several interventions without success, or who have complex emotional relationships with food. Drug therapy can be considered, combined with behavioural, dietary and physical activity approaches, if these have been unsuccessful when used alone [1]. Aneurin Bevan MDWMC is classed as a Level 3 service, and, here, this remit translates to individual, one-to-one appointments with a Consultant Physician, Specialist Nurses, a Cognitive Behavioural Psychotherapist, a Registered Dietitian, and a Physiotherapist, should the latter three services be deemed necessary.

### 1.6. The 1:1 Multidisciplinary Weight Management Clinic

Aneurin Bevan MDWMC has approximately 150 patients in active treatment. The treatment period extends from between 6 and 24 months and is individually tailored. Referrals are received from GPs, Physicians, Specialist Nurses, Practice Nurses, Psychiatrists and surgeons. Referral Criteria are age 16 years and above and a BMI of 35 Kg/m^2^ and above. The clinic is held weekly at Aneurin Bevan Hospital and the waiting list currently stands at approximately 400 patients.

Once patients attend the clinic, a nurse takes basic measurements, such as weight, BMI, blood pressure, and waist circumference. Each patient then sees the doctor and, if applicable, a mood assessment is conducted. Typically, a patient will return for a second appointment after four to six weeks where the patient and doctor will decide whether additional 1:1 appointments with the other health professionals are appropriate. Patients return to see the doctor at four to six week intervals, in addition to any other appointments with other multidisciplinary team (MDT) members, and will see the clinic nurse to be weighed and measured prior to any of these appointments.

### 1.7. Current Literature

Following a literature search on Google, Google scholar, Pub Med, and Cochrane Library, evidence was available to demonstrate effectiveness of such treatment programmes. Indeed, studies specifically on Aneurin Bevan MDWMC have demonstrated its efficacy [11,12,13]. This service evaluation was also interested in exploring the reasons for any successes. With exception of directly related studies, most published evidence referred to multidisciplinary clinics delivering services in a group setting—this service evaluation was particularly interested in investigating the value of the individual 1:1 approach.

### 1.8. Objectives

The objectives of this study are to determine both physiological benefits and qualitative information, namely patient satisfaction, associated with the service. We intend to examine, therefore: Qualitative and Quantitative outcomes of a 1:1 Multidisciplinary weight management clinic.

## 2. Methodology

This service evaluation took the form of semi-structured interviews with patients attending the MDWMC at Aneurin Bevan Hospital, Wales, between December 2011 and December 2012. Data were collected during patient interviews, and recorded on a data collection sheet (Appendix B).

A questionnaire used to facilitate interviews was devised by key MDT members. As summarised in Table 1, 26 key items were assessed. Specialist advice in construction of the questionnaire was sought from Picker Institute Europe, a not-for-profit organisation that specialises in designing patient experience surveys. Permission to conduct the study was granted by Aneurin Bevan Health Board Research Risk Review Committee.

**Table 1:** A summary of the 26 key items audited (and the question number on our data collection form each refers to).

Key Items for Auditing	Question Number on Data Collection Form
Treatment length	1
Weight Loss	2
Preference of group or individual appointments	3
Activity levels	4a
Confidence levels	
Diet improvement	
Usefulness of meeting Nurse	4b
Usefulness of meeting Doctor	
Usefulness of meeting Dietitian	
Usefulness of meeting Counsellor	
Usefulness of meeting Physiotherapist	
Encouragement to lead healthy lifestyle	5
Health improvement	6
Medication change—blood pressure	7
Medication change—asthma	
Medication change—painkillers	
Medication change—cholesterol	
Medication change—diabetes/insulin	
Medication change—angina	
Medication change—antidepressants	
Medication change—Other/over-the-counter	
Recommendation of clinic to others	8
Employment status	9a
Reduction in sick days	9b
Suggestions for improvement of clinic	10
Any further comments	11

### 2.1. Pilot Study

A pilot study of 10 interviews was conducted during on Thursday 1 and Thursday 8 December 2011. Changes to the interview questionnaire involved simplification of the fixed response boxes and separation of some questions to simplify the process.

### 2.2. Scope

Throughout this service evaluation:

- All active patients attending the MDWMC were eligible for interview—time since referral was not cause for exclusion.
- New patients visiting clinic for the first occasion were excluded from interview.
- Discharged patients, patients lapsing at six months, and patients who did not attend clinic were not interviewed.

### 2.3. Data Collection

To produce meaningful results a sample size of 180 interviews was achieved. Interviews were conducted in person when patients attended the clinic, and by telephone. All were conducted in private by a medical student who was introduced as independent to the clinic team, and who asked questions and recorded patient responses. All active patients were required, for the interview, to achieve a target sample size, though new patients visiting the clinic for the first time were excluded as they had not yet commenced treatment. All patients were asked for their consent in order to take part, before the interview, were assured their responses would remain confidential, and were assured that their identifiable responses were not shared with clinic staff. One medical student carried out patient interviews between December 2011 and April 2012, and a different medical student conducted interviews between October and December 2012.

In order to prevent duplication of patient data, patient details were recorded and cross-matched during data analysis. All patient data was protected using an encrypted electronic database. Patient details were deleted once data analysis was completed.

### 2.4. Data Analysis

Data were tabulated and analysed using pivot tables within Microsoft Excel by the second medical student. A thematic analysis of free text responses was carried out by the second medical student to develop detailed codes, and individual quotes were identified for illustrative purposes. On occasion, some difficulty was encountered by the second student in interpreting free text responses documented by the first student. Statistical analysis was not conducted as data was not deemed appropriate for available testing [14].

## 3. Results

A total of 180 patients were interviewed—11 patients declined to participate giving a response rate of 94%. Of these, 131 were female, 49 were male and ages ranged between 19 and 74.

### 3.1. How Long Have You Been Attending the Clinic?

Interviewed patients were more-or-less evenly spread across the clinic’s maximum 24-month attendance period (Table 2).

**Table 2:** Length of attendance of interviewed patients at MDWMC.

Category	<3 Months	3–6 Months	6–12 Months	>12 Months	No Response	Total
No. patients	43	27	56	53	1	180

### 3.2. Have You Lost Weight since Attending the Clinic?

Of the 180 patients interviewed, 109 had been attending the clinic for longer than six months: 95% (*n* = 104) of these patients noticed weight loss (Table 3).

**Table 3:** Weight loss over various lengths of attendance at MDWMC.

Category	<3 Months	3–6 Months	6–12 Months	>12 Months	No Response	Total
Lost weight	26	23	54	50	1	154
Not lost weight	17	4	2	3	0	26

Baseline weights were available from clinic records for 124 interviewed patients and percentage weight loss was calculated. 73% achieved at least 5% weight loss (Table 4).

**Table 4:** Percentage of total body weight lost.

% Weight Loss	5%	10%	Total
*n*	45	46	124
%	36	37	73

### 3.3. If You Were Offered a Choice of Appointment Type, Which Would You Choose—Individual Appointment or Group? Please Comment on Your Choice

Of all interviewed patients, 176 responded to part one: 88% of respondents (*n* = 155) preferred individual appointments and 12% (*n* = 21) preferred group appointments.

Respondents were asked to give reason for their appointment preference: only seven declined to give any explanation—five of those indicating individual preference, and two of those indicating preference for groups. Patients were not restricted by the number of reasons they could give for their choice: 74% (*n* = 125) gave one reason, 24% (*n* = 41) patients offered two reasons, and 2% (*n* = 3) patients offered three.

#### Preference for Individual Appointments

**Figure 1:**
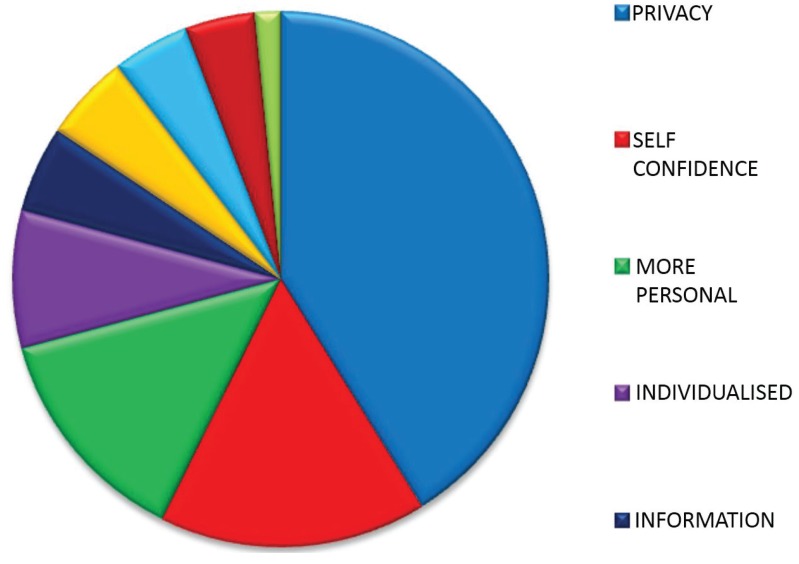
Proportions of respondents giving various reasons for preferring individual appointments.

The 88% (*n* = 155) of patients who indicated a preference for individual responses gave a total number of 192 reasons for their choice. “Privacy”, “self-confidence”, “personal issues”, and needing “more personal” relationships with healthcare professionals accounted for nearly 80% of reasons (Table 5, Table 6 and Figure 1).

**Table 5:** Number and percentage of respondents giving various reasons for preferring individual appointments.

Category	*n*	%
Privacy	79	41
Self Confidence	31	16
More Personal	26	14
Personal Issues	16	8
Individualised Information	10	5
Both	10	5
Dislikes Groups	9	5
No Benefits	8	4
Takes Less Time	3	2
Total	192	100

**Table 6:** Explanation of reasons respondents gave for preferring individual appointments.

Category	Meaning	Patient Quotes
Privacy	Desire a private arena to discuss their weight	“It’s more private”
		“My weight is a private issue”
		“I prefer to talk in private as I suffer from panic attacks”
Self Confidence	Feel uncomfortable discussing weight in front of other patients	“I’m embarrassed to talk about my weight in a group”
		“I wouldn’t have the confidence to speak up in a group”
More Personal	Prefer to establish a personal relationship with healthcare professional	“I like building up a rapport with the clinicians”
		“As I get to know the clinicians I feel I can open up more”
Personal Issues	Wish to discuss issues which they feel are personal to them	“My weight is a personal issue and I need the full attention of a healthcare professional”
		“You can focus on personal issues in an individual appointment”
Individualised Information	Seek information which they feel needs tailoring to their individual situation	“I prefer talking to clinicians”
		“Hearing the information face to face makes it seem more important”
Both	Would like to attend individual appointments and groups	“It would be good to attend a group as well later on”
		“I’d like to attend a group as well to help keep me motivated on a more regular basis”
Dislikes Groups	Dislike group settings	“I don’t like groups”
No Benefits	Have not benefitted from groups in past	“I haven’t benefitted from groups in the past”
		“Individual appointments are more motivating”
Takes Less Time	Individual appointments take less time to address issues	“It takes less time”

### 3.4. Lifestyle Change

**Figure 2:**
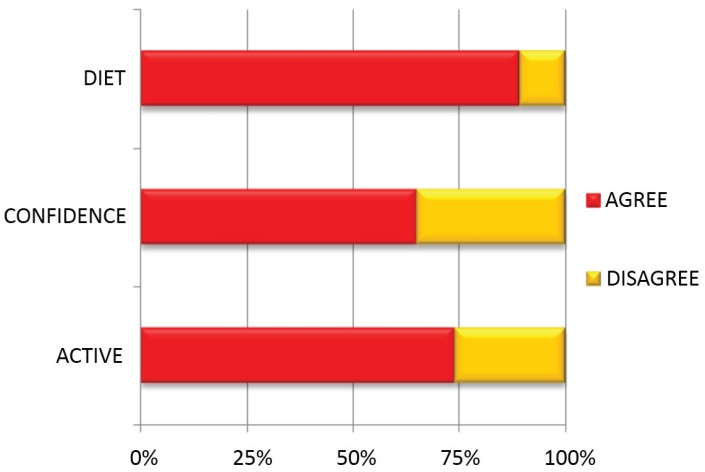
Percentage of respondents having made lifestyle changes.

The majority of patients made positive lifestyle changes since attending the clinic: 74% (*n* = 133) of patients said they had become more active, 65% (*n* = 116) of patients said they had become more confident and 89% (*n* = 161) of patients said they had improved their diet (Figure 2, Table 7).

### 3.5. Efficacy of MDT Consultations

Patients were asked to indicate how useful their consultations were with individual team members. Not all patients saw each member of the MDT (Table 8).

**Table 7:** Number of respondents having made lifestyle changes.

Category	Agree (*n*)	Disagree (*n*)	Total (*n*)
Improved Diet	161	19	180
More Confident	116	64	180
More Active	133	47	180

**Table 8:** Number and proportion of patients seeing each healthcare professional.

No. of Patients Who Saw Team Member	Nurse	Doctor	Dietitan	Counsellor	Physiotherapist	Max
***n***	180	180	110	106	23	**180**
**%**	100	100	61	59	13	**100**

**Figure 3:**
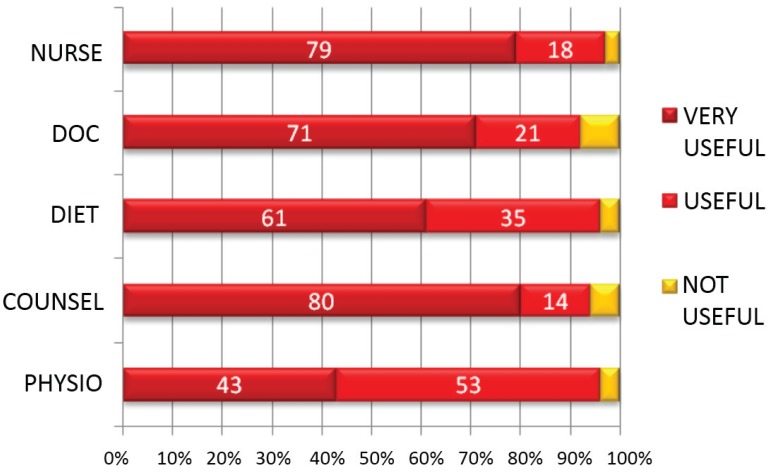
Percentage of respondents finding consultations with each healthcare professional very useful, useful and not useful.

Over 90% of patients who saw each team member found the consultations useful (Figure 3, Table 9).

**Table 9:** Percentage of respondents finding consultations with each healthcare professional useful.

Category	Very Useful (%)	Useful (%)	Not Useful (%)
Nurse	79	18	3
Doctor	71	21	8
Dietitian	61	35	4
Counsellor	80	14	6
Physio	43	53	4

### 3.6. Since Attending the Clinic Have You Felt Encouraged to Lead a Healthier Lifestyle, Yes or No? Please Explain

Of all interviewed patients, 174 responded to the part one: 93% of respondents (*n* = 162) stated “Yes” they felt encouraged to lead a healthier lifestyle and 7% (*n* = 12) stated “no” they did not.

Respondents to part one were asked to give reason for their response: only four declined to give any explanation—two of those responding “yes”, and two of those responding “no”. Patients were not restricted by the number of reasons they could give for their choice: 63% (*n* = 107) gave one reason, 29% (*n* = 50) patients gave two, and 8% (*n* = 13) patients gave three.

“Yes” Encouraged to lead a healthier lifestyle.

The 93% (*n* = 162) of patients who stated that “Yes” they felt encouraged to lead a healthier lifestyle gave a total number of 235 reasons for their choice.

**Figure 4:**
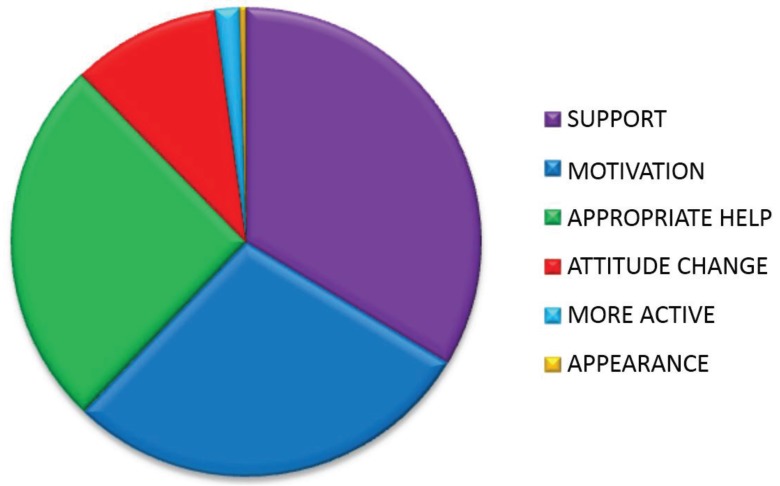
Proportion of reasons given by patients for feeling encouraged to lead a healthier lifestyle.

Reasons given for feeling encouraged to lead a healthier lifestyle are tabulated and explained in Figure 4, Table 10 and Table 11.

**Table 10:** Number and percentage of reasons given by patients for feeling encouraged to lead a healthier lifestyle.

Reason	*n*	%
Support	79	34
Motivation	67	29
Appropriate help	60	26
Attitude change	24	9
More active	4	1
Appearance	1	1
Total	235	100

**Table 11:** Explanation of reasons given by patients for feeling encouraged to lead a healthier lifestyle.

Category	Meaning	Patient Quotes
Support	Support given by team	“For the first time I feel I’m being listened to”
		“The teams’ attitudes create a positive environment”
Motivation	Motivated by team	“I feel motivated in the right way”
		“Regular appointments keep motivation up”
Appropriate help	Receiving appropriate information	“Being told 1:1 by a clinician the consequences of not addressing my weight made me understand the seriousness of the issue”
		“Seeing the dietitian to find out where I was going wrong”
Attitude change	A change of attitude has allowed behaviour change	“I feel more mentally strong”
		“Sessions with the counsellor have helped me feel I can do it”
More active	Weight loss enabled more activity	“I’m able to move more because I’ve lost weight”
Appearance	Wanted to look different	“I wanted to change the way I looked”

### 3.7. Since Attending the Clinic Have You Felt Your Health Has Improved Yes or No? Please Explain

All interviewed patients responded to part one: 69% (*n* = 124) patients answered that “Yes” they felt their health had improved since attending the clinic, and 31% (*n* = 56) replied that “No” they felt their health had not improved.

Patients were then asked to give reason for their response: only eight declined to give any explanation—five of those responding “yes”, and three of those responding “no”. Patients were not restricted by the number of reasons they could give for their choice: 48% (*n* = 82) gave one reason, 41% (*n* = 71) patients gave two reasons, and 11% (*n* = 19) patients gave three.

“Yes” Health improved.

The 69% (*n* = 124) of patients who stated that “Yes” they felt their health had improved gave a total number of 202 reasons for their choice.

**Figure 5:**
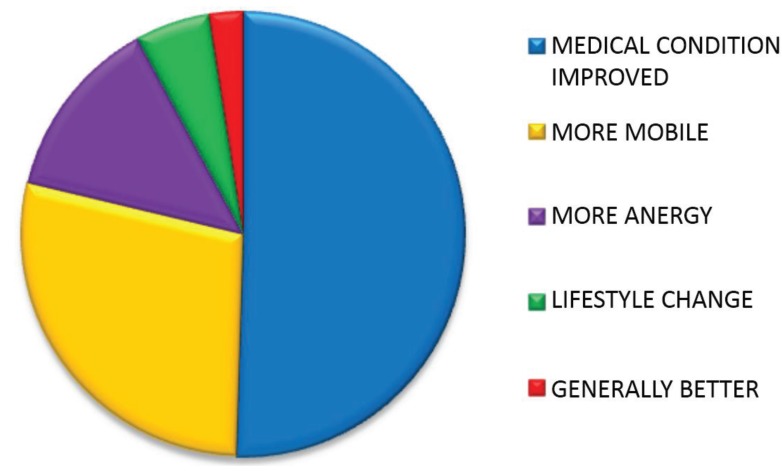
Proportion of reasons given by patients for feeling their health had improved.

Reasons given for feeling health had improved are tabulated and explained in Figure 5, Table 12 and Table 13.

**Table 12:** Number and percentage of reasons given by patients for feeling their health had improved.

Reason	*n*	%
Medical condition	102	51
More mobile	57	28
More energy	27	13
Lifestyle change	11	5
Generally better	5	3
TOTAL	202	100

**Table 13:** Explanations of reasons given by patients for their health had improved.

Category	Meaning	Patient Quotes
Medical condition	Improvement in medical condition	“Resolves sleep apnoea” “I no longer need insulin”
More mobile	More mobile	“I can get about more ” “I am more active”
More energy	More energy	“I am less tired” “I have more energy”
Lifestyle change	A non-medical improvement to life	“I am less stressed” “I have lost weight”
Generally better	Sense of feeling “better”	“I just generally feel better”

“No” Health not improved.

The 31% (*n* = 56) patients who stated that “No” they felt their health had not improved gave a total number of 60 reasons for their choice.

**Figure 6:**
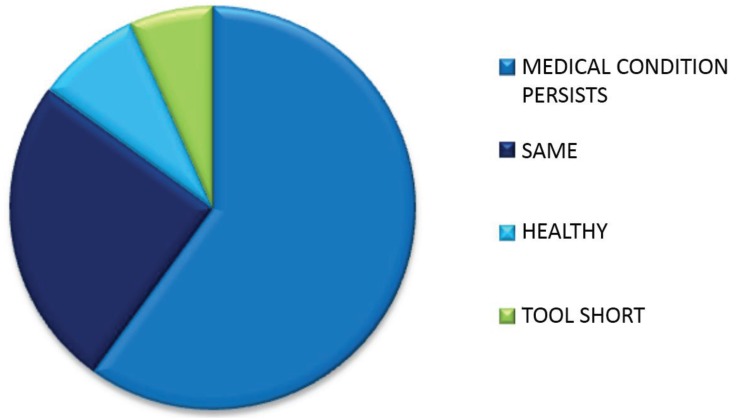
Proportion of reasons given by patients for feeling their health had not improved.

Reasons given for feeling health had improved are tabulated and explained in Figure 6, Table 14 and Table 15.

**Table 14:** Number and percentage of reasons given by patients for feeling their health had not improved.

Reason	*n*	%
Medical condition	36	60
Health same	15	25
Already healthy	5	8
Attendance too short	4	7
Total	60	100

**Table 15:** Explanation of reasons given by patients for feeling their health had not improved.

Category	Meaning	Patient Quotes
Medical condition	No improvement in medical condition	“My blood pressure is still high” “My joints are still ainful”
Health same	Feels health is the same	“I don’t feel any more healthy” “My health is the same”
Already healthy	Health was good before	“I wasn’t unhealthy before”
Attendance too short	Time attending clinic is too short to feel benefits	“It’s too early to feel healthier yet”

The most common reason patients gave for feeling that their health had improved was related to an improvement of a health condition. Table 16 shows the most common medical conditions that improved since patients had been attending the clinic, and their percentage of all reasons given for indicating improved health.

**Table 16:** The most common medical conditions that improved since patients had been attending the clinic, and their percentage of all reasons given for indicating improved health.

Medical Condition	Yes (*n*)	% of All Reasons Given for Indicating Improved Health
Less Short Of Breath	41	20.5
Better Sleep	13	6.5
Better Controlled Blood Pressure	12	6
Less Joint Pain	11	5.5
Better Mental Health	6	3
Fewer Symptoms of Diabetes	5	2.5
Other	14	7
Totals	102	51

### 3.8. If You Were Taking Medication for the Following Medical Conditions—Have They Been Decreased since Attending the Clinic?

**Figure 7:**
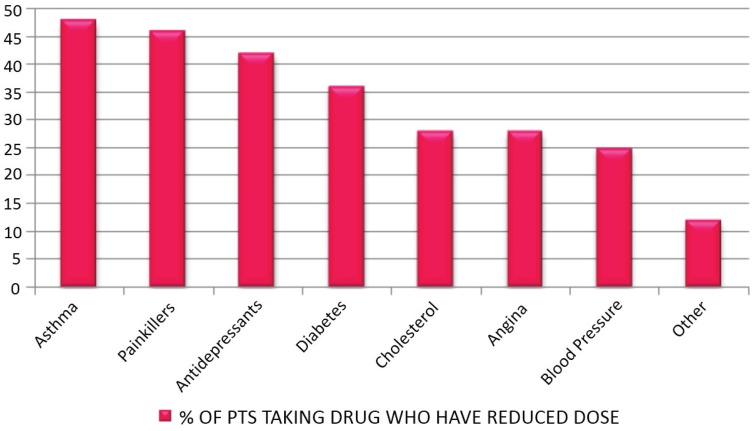
Percentage of patients taking obesity-related drugs who have reduced dose.

Just under half of patients who took asthma medication decreased their dose. Over 40% of patients taking painkillers reduced their dose since attending the clinic. Forty percent of patients taking antidepressants showed a reduction in dosage. Figure 7 and Table 17 show the percentage of patients taking specified medication who reduced their dose since attending the clinic.

### 3.9. Would You Recommend the Clinic to Others?

**Figure 8:**
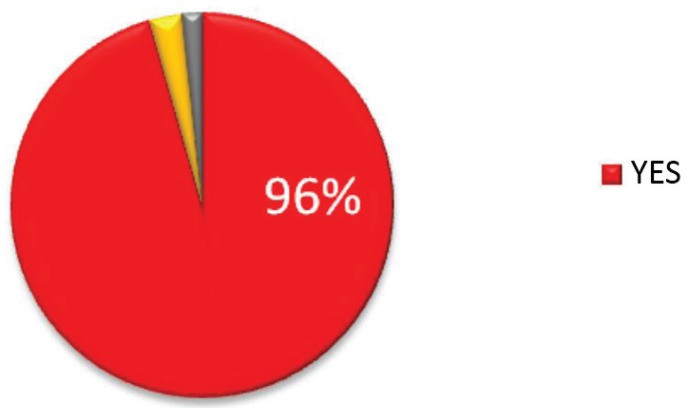
Proportion of patients who would recommend the clinic.

The proportion of patients who stated they would recommend the clinic to others was 96% (*n* = 172), 3% (*n* = 5) said they would not, and 2% (*n* = 3) patients did not respond (Figure 8).

**Table 17:** Number and percentage of patients taking obesity-related drugs who have reduced dose.

Medication Decrease	*n*	Total *n*	%
Asthma	23	48	48
Painkillers	53	116	46
Antidepressants	27	65	42
Diabetes	14	39	36
Cholesterol	16	57	28
Angina	5	18	28
Blood Pressure	22	88	25
Other	3	25	12

### 3.10. Are You Employed? If Yes Are You Taking Less Time Off Because of Ill Health Since Attending the Clinic? Yes/No

Of the 69 patients who were employed, 30% (*n* = 21) said they were taking less time off work due to ill health since attending the clinic. Only seven patients did not respond.

### 3.11. Improvements

**Figure 9:**
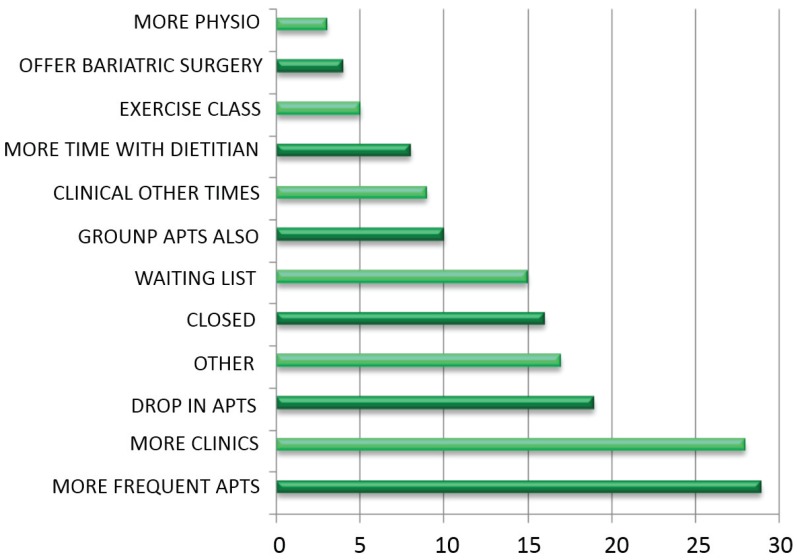
Number times each improvement was suggested.

Patients were asked to suggest improvements that the clinic could make. The number of suggestions was not restricted (Figure 9, Table 18 and Table 19).

**Table 18:** Number of times each improvement was suggested.

Improvement	*n*
More physio	3
Surgery	4
Exercise class	5
Dietitian	8
Other times	9
Groups also	10
Waiting List	15
Closer	16
Other	17
Drop In apts.	19
More clinics	28
More frequent apts.	29

### 3.12. Comments

**Figure 10:**
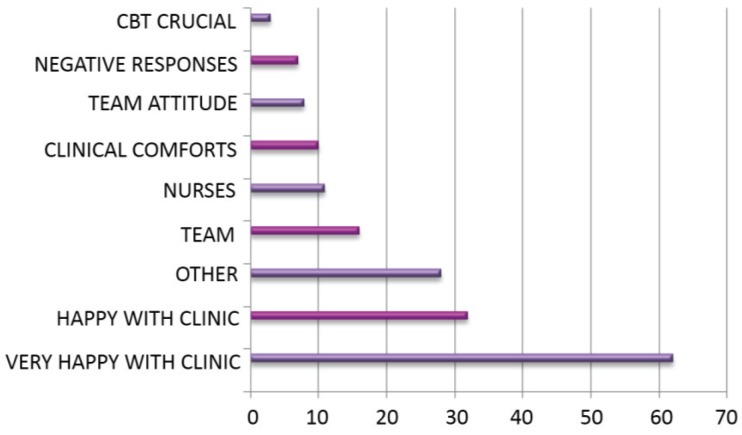
Number times each comment was made.

Patients were asked to make any further general comments. The number of suggestions was not restricted (Figure 10, Table 20 and Table 21).

**Table 19:** Explanation of suggested improvements.

Category	Meaning	Patient quotes
More physio	More time with physiotherapist	“I’d like more demonstrations from the physiotherapist”
Surgery	Offer bariatric surgery	“Include bariatric surgery at the clinic”
Exercise class	Offer an exercise class	“The clinic could so with an exercise class liked to it” “Include referral to GP exercise scheme”
Dietitian	More time with dietitian	“I’d like more time with the dietitian” “Provide more dietitians”
Other times	Run clinic at other times	“Offer clinics in the evenings” “Offer appointments at more flexible times”
Groups also	Offer group appointments also	“Run group sessions also to motivate in-between appointments” “Offer group sessions for more frequent monitoring”
Waiting List	Reduce waiting list	“Reduce time required to wait on the list for referral to the clinic”
Closer	Run clinics close to patients’ homes	“It would be easier if I didn’t have to travel so far”
Other	Other suggestions	“Reduce walking distance from car-ark to clinic”
Drop In apts.	Offer drop in appointments	“Offer drop in appointments for weigh-ins”
More clinics	Run more clinics	“Provide more clinics”

**Table 20:** Number of times each comment was made.

Comment	*n*
CBT crucial	3
Negative	7
Team attitude	82
Clinic comforts	10
Nurses	11
Team	16
Other	28
Happy with clinic	32
Very happy with clinic	62

**Table 21:** Explanation of comments.

Category	Meaning	Patient Quotes
CBT crucial	*Counsellor session is crucial*	“The sessions provided by the counsellor are crucial”
Negative	*Negative responses*	“I’m really unhappy with everything”
Team attitude	*Attitude of team members is significant*	“The team are very welcoming” “The team’s attitude is great”
Clinic comforts	*Clinic provides a comforting environment*	“The clinic is a comfort zone” “The clinic is a lifeline”
Nurses	*Specific mention of the nurses*	“The nurses are great” “I have a great rapport with the nurses”
Team	*Specific mention of the team members as a whole*	“The staff are great. I really look forward to going to clinic” “The team are great”
Other	*Other comments*	“The clinic runs smoothly ”
Happy with clinic	*Happy with clinic*	“I am happy with everything”
Very happy with clinic	*Very happy with clinic*	“I am very happy with the clinic—I would be bedridden without it”

## 4. Discussion

### 4.1. Summary of Main Findings and Comparisons with Literature

The data shows the MDWMC is effective in achieving weight loss: 95% of patients interviewed had lost weight. Two-thirds of those who had not lost weight had been attending the clinic for less than three months and this may explain the non-losses. Additionally, data was not collected to assess whether any weight *gain* had occurred with the non-weight losers—a halt in weight gain may be considered successful treatment. For the 124 patients for whom baseline data was available, 73% had achieved weight loss of at least 5% of initial body weight with 37% achieving 10% or more: this meets the NICE guidelines target of achieving 5%–10% loss of original weight [10]. This exceeds results from a previous effectiveness study of the Aneurin Bevan MDWMC, where 51% of the population achieved loss of 5% initial body weight or more [12], although patients in this study were only monitored for six months—our data includes patients from less than six months to up to 24 months of attendance. It must be noted that this study’s data includes weight losses reported by patients so it is possible there may be some discrepancy between “reported” and “actual” losses.

The tailored treatment, which is afforded by the 1:1 appointment system, is strongly supported, with 88% of patients opting for individual appointments if given a choice between 1:1 or group treatment. The reasons for this, elicited by the accompanying free text, exposed patients’ inability to fully address their weight management issues in groups—nearly 80% of reasons given broadly addressed this with recurring themes of issues with “privacy”, “self-confidence”, “personal issues”, and needing “more personal” relationships with healthcare professionals. While comparative research is not available, this preference has been echoed in a study where adolescents and parents attending a MDWMC felt individual sessions addressing their personal concerns were viewed as important [15]. The individual appointments are a unique feature of the 1:1 MDWMC and are not routinely offered as part of any other treatments contained in The All Wales Obesity Pathway.

Patients reported positive lifestyle changes: 74% increasing activity, 65% increasing confidence, and 89% improving diet. These figures are already favourable, but some additional positive responses may have been masked by the question wording: some patients volunteered they were not lacking in confidence or were already active prior to attending clinic and so could not answer that attendance at MDWMC had encouraged either of these. For patients who have previously failed to achieve weight loss in group settings, this finding may indicate an attempt to finally change lifestyle habits. Certainly it may be linked to the >90% of interviewees who reported that they find each individual consultation with healthcare professionals useful: recent guidelines issued by the UK’s Royal College of Physicians list all the MDT members found at Aneurin Bevan MDWMC as recommended participants in any MDWMC, and thus echoes the findings in our report [16]. Reasons given for changing lifestyle habits may also be explained by the same reasons 93% of respondents gave for feeling encouraged to lead a healthier lifestyle: 34% were due to support, 29% due to motivation, and 26% were due to appropriate education. A long-term follow-up of lifestyle change would indicate whether these behaviour changes become habitual over the long term, as has been demonstrated elsewhere [17]. “Attendance at MDWMC helps to improve health” is agreed with by 69% of respondents. Half the reasons given are due to experiencing reductions in symptoms of obesity-related diseases. A collective 41% say this is due to an increase in mobility and having more energy. Importantly, of the one-third of patients who stated they felt their health had not improved did not give a deterioration of their health as a reason: the reasons were mostly that they suffered from symptoms of a medical condition which was not always related to their obesity and which could not be assisted by weight loss. Other reasons for non-improvement were that patient health had stayed the same or they already considered themselves to be in good health prior to attending clinic.

A reduction in symptoms of obesity-related diseases coupled with reported reductions in dose of obesity-related medications, has significant cost-saving implications: up to 48% of patients taking painkillers, antidepressants, and drugs for asthma, diabetes, cholesterol, angina, and blood pressure reduced their doses. Although the reported nature of responses makes the estimation less accurate, this could be assessed more accurately by further study. These figures make some headway into estimating potential cost savings from fewer prescriptions, visits to primary care professionals, and hospital admissions. It is estimated that, of all drugs prescribed in primary care in the UK, 25% of costs are attributable to overweight and obesity, and obesity more than doubled prescribing in primary care in most drug categories [7]. As a result of obesity, it is estimated that 64% additional contacts are made with the GP per year, equating to approximately nearly 12% of all costs in Wales [7]. In an economic sense, it is also encouraging that 30% of employed patients reported a reduction of days taken off work due to sickness, given that, in England, obesity is estimated to account for 18 million sick days per year [18].

Patients are overridingly happy with their treatment at the clinic, and, with 96% agreeing with the statement, would almost unanimously recommend it to others. When asked to volunteer any other information, satisfaction with the MDWMC is reiterated in the vast majority of responses, followed by the appreciation of team members in shaping patient experience. When asked what improvements could be made, patients overwhelmingly ask for more access to the clinic and its related services, which makes for a strong message when considering expenditure related to patient needs.

### 4.2. Strengths and Limitations of the Study

As already mentioned, for the data, which assessed physiological measures, this study relied on the accuracy of patient reporting. While this may differ from actual measures, this evaluation points to likely outcomes and may be more accurately assessed by further study.

All data in this study was collected by patients in active attendance at MDWMC. No attempt was made to assess the attrition rate and interviewing patients who either self-discharged, were lost to six-month follow-up, or did not attend clinic would give a more balanced view of how broadly successful the clinic is in serving its eligible population.

The methods of data collection must be considered in assessing accuracy of responses: the two interviewers were of different age and sex and these elements have been known to cause bias, but responses did not show significant difference in this study. It must also be considered that, while the interviewers were introduced as independent medical students, they may be thought of as part of the healthcare team by patients and, thus, may deter patients from giving true responses. Patients also might not trust the confidentiality and anonymity statements issued and give more positive responses in order to protect their treatment.

### 4.3. Suggestions for Future Work

The wealth of information held by Aneurin Bevan 1:1 Multidisciplinary Clinic could be more extensively employed to validate the propositions made by this study. The main challenge for further work is in collecting the data, as, to date, the clinic has no funded administrative support and relies on good-willed clinic nurses to do so. More physiological measurements, such as pre- and post-treatment lipids and inflammatory markers, would be interesting to compare. Cross-referencing of data could explore any predictions for successes of treatment in certain patient groups, and, therefore, lead to higher efficacy through refining the referral criteria. A longitudinal study would assess the lasting value of the clinic, which gives the ultimate cost-saving return. It would also be interesting to attempt to compare qualitative and quantitative benefits of the various obesity-reducing services offered at various levels of the All Wales Obesity Pathway. Finally, it would be useful to enquire whether any patients taking obesity-reducing drugs reported any side-effects to treatment.

## 5. Conclusions

A 1:1 Multi-disciplinary Weight Management Clinic provides value in reducing obesity, symptoms of obesity-related diseases, and associated costs to healthcare services. It improves perceived health, health-related behaviours, and fitness to work. Patients commented that it is an intervention method that they like.

## Figures and Tables

**Figure A1:**
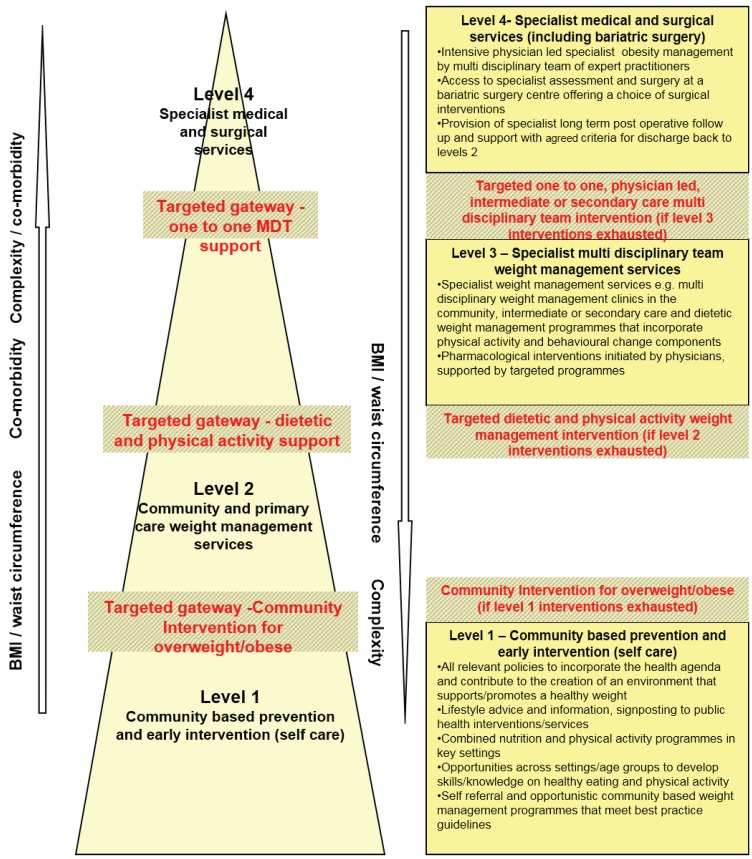
The All Wales Obesity Pathway.

## Appendix B. Questionnaire for Patient Interviews. Aneurin Bevan Specialist Weight Management Clinic

Please would you take a few minutes of your time to answer some questions regarding the clinic. Any information used in this patient survey will be held in complete confidence.

(1) How long have you been attending the clinic? <3 months/3–6 months/6–12 months/>12 months
(2) Have you lost weight since attending the clinic? Yes/No
  If yes how much weight have you lost?
(3) If you were offered, on referral to the clinic, a choice of appointments type which would you choose?
  (Please circle) Individual appointment OR Group
  Please include comments
(4a) Please indicate how much you agree with the following statements
  **Table:**
  Statements	Strongly Agree	Agree	Disagree	Strongly Disagree
  I have become more active since attending the clinic				
  I have become more confident since attending the clinic				
  I have improved my diet since attending the clinic				
(4b) How useful was your meeting with the following?
  **Table:**
  Professional	Very Useful	Useful	Not very Useful	Did Not Meet
  Nurse				
  Doctor				
  Dietitian				
  Counsellor				
  Physiotherapist				
(5) Have you felt encouraged to lead a healthier lifestyle?—Yes/No
  Please explain
(6) Since attending the clinic have you felt your health has improved?—Yes/No
  Please explain
(7) If you were taking medication for the following medical conditions—have they been decreased since attending the clinic?
  **Table:**
  Medication	Yes—Decreased	No—The Same	Not Applicable
  Blood Pressure			
  Ashma			
  Painkillers			
  Cholesterol			
  Diabetes Tablets/Insulin			
  Angina			
  Antidepressants			
  Other Medication e.g., over the counter			
(8) Would you recommend the clinic to others?—Yes/No
(9) Are you employed? Yes/No
  If yes are you taking less time off because of ill health since attending the clinic? Yes/No
(10) If you could make any improvements to the clinic, what would they be?

Any other comments

Thank you for your help in completing this form
